# Supplementary material for: HIVprotI: an integrated web based platform for prediction and design of HIV proteins inhibitors
Source: J Cheminform. 2018 Mar 9;10:12. doi: 10.1186/s13321-018-0266-y (PMC5845081; doi:10.1186/s13321-018-0266-y)
Supplement: Supplementary file 2 — Additional file 2. Source code of HIVProtI web server. [file 13321_2018_266_MOESM2_ESM.zip › HIVprotI_Source-code/hivprotI/weka-3-6-6/doc/weka/classifiers/functions/pace/PaceMatrix.html]

PaceMatrix


---


|  |  |  |
| --- | --- | --- |
| **Overview** \| **Package** \| **Class** \| **Tree** \| **Deprecated** \| **Index** \| **Help** \| **Weka's home** | |  |
| **PREV CLASS**   NEXT CLASS | **FRAMES**    **NO FRAMES**     **All Classes** |  |
| SUMMARY: NESTED \| FIELD \| CONSTR \| METHOD | DETAIL: FIELD \| CONSTR \| METHOD |  |


---


## weka.classifiers.functions.pace Class PaceMatrix

```
java.lang.Object
  weka.core.matrix.Matrix
      weka.classifiers.functions.pace.PaceMatrix
```

**All Implemented Interfaces:**: java.io.Serializable, java.lang.Cloneable, RevisionHandler

---

``` public class PaceMatrix extends Matrix ```

Class for matrix manipulation used for pace regression.

REFERENCES

Wang, Y. (2000). "A new approach to fitting linear models in high
dimensional spaces." PhD Thesis. Department of Computer Science,
University of Waikato, New Zealand.

Wang, Y. and Witten, I. H. (2002). "Modeling for optimal probability
prediction." Proceedings of ICML'2002. Sydney.

**Version:**
:   $Revision: 1.6 $

**Author:**
:   Yong Wang (yongwang@cs.waikato.ac.nz)

**See Also:**: Serialized Form

---

**Constructor Summary**

`PaceMatrix(double[][] A)`
  
          Construct a PACE matrix from a 2-D array.

`PaceMatrix(double[][] A,
int m,
int n)`
  
          Construct a PACE matrix quickly without checking arguments.

`PaceMatrix(double[] vals,
int m)`
  
          Construct a PaceMatrix from a one-dimensional packed array

`PaceMatrix(DoubleVector v)`
  
          Construct a PaceMatrix with a single column from a DoubleVector

`PaceMatrix(int m,
int n)`
  
          Construct an m-by-n PACE matrix of zeros.

`PaceMatrix(int m,
int n,
double s)`
  
          Construct an m-by-n constant PACE matrix.

`PaceMatrix(Matrix X)`
  
          Construct a PaceMatrix from a Matrix


| **Method Summary** | |
| --- | --- |
| `void` | `backward(PaceMatrix b, IntVector pvt, int ks, int k0)`             Backward ordering of columns in terms of response explanation. |
| `PaceMatrix` | `cbind(PaceMatrix b)`             Returns a new matrix which binds two matrices with columns. |
| `java.lang.Object` | `clone()`             Clone the PaceMatrix object. |
| `double` | `columnResponseExplanation(PaceMatrix b, IntVector pvt, int j, int ks)`             Returns the squared ks-th response value if the j-th column becomes the ks-th after orthogonal transformation. |
| `void` | `forward(PaceMatrix b, IntVector pvt, int k0)`             Forward ordering of columns in terms of response explanation. |
| `double[]` | `g1(double a, double b)`             Constructs the Givens rotation |
| `void` | `g2(double[] cs, int i0, int i1, int j)`             Performs the Givens rotation |
| `DoubleVector` | `getColumn(int j)`             Return a DoubleVector that stores a column of the matrix |
| `DoubleVector` | `getColumn(int i0, int i1, int j)`             Return a DoubleVector that stores some elements of a column of the matrix |
| `java.lang.String` | `getRevision()`             Returns the revision string. |
| `double[]` | `h1(int j, int k)`             Constructs single Householder transformation for a column |
| `void` | `h2(int j, int k, double q, PaceMatrix b, int l)`             Performs single Householder transformation on one column of a matrix |
| `boolean` | `isEmpty()`             Check if the matrix is empty |
| `int` | `leastExplainingColumn(PaceMatrix b, IntVector pvt, int ks, int k0)`             Returns the index of the column that has the smallest (squared) response, when the column is moved to become the (ks-1)-th column. |
| `void` | `lsqr(PaceMatrix b, IntVector pvt, int k0)`             QR transformation for a least squares problem |
| `void` | `lsqrSelection(PaceMatrix b, IntVector pvt, int k0)`             QR transformation for a least squares problem |
| `static void` | `main(java.lang.String[] args)`             for testing only |
| `double` | `maxAbs()`             Returns the maximum absolute value of all elements |
| `double` | `maxAbs(int i0, int i1, int j)`             Returns the maximum absolute value of some elements of a column, that is, the elements of A[i0:i1][j]. |
| `double` | `minAbs(int i0, int i1, int column)`             Returns the minimum absolute value of some elements of a column, that is, the elements of A[i0:i1][j]. |
| `int` | `mostExplainingColumn(PaceMatrix b, IntVector pvt, int ks)`             Returns the index of the column that has the largest (squared) response, when each of columns pvt[ks:] is moved to become the ks-th column. |
| `DoubleVector` | `nnls(PaceMatrix b, IntVector pvt)`             Solves the nonnegative linear squares problem. |
| `DoubleVector` | `nnlse(PaceMatrix b, PaceMatrix c, PaceMatrix d, IntVector pvt)`             Solves the nonnegative least squares problem with equality constraint. |
| `DoubleVector` | `nnlse1(PaceMatrix b, IntVector pvt)`             Solves the nonnegative least squares problem with equality constraint. |
| `void` | `positiveDiagonal(PaceMatrix Y, IntVector pvt)`             Sets all diagonal elements to be positive (or nonnegative) without changing the least squares solution |
| `static Matrix` | `randomNormal(int m, int n)`             Generate matrix with standard-normally distributed random elements |
| `PaceMatrix` | `rbind(PaceMatrix b)`             Returns a new matrix which binds two matrices together with rows. |
| `void` | `rsolve(PaceMatrix b, IntVector pvt, int kp)`             Solves upper-triangular equation |
| `void` | `setColumnDimension(int columnDimension)`             Set the column dimenion of the matrix |
| `void` | `setMatrix(double[] v, boolean columnFirst)`             Set the whole matrix from a 1-D array |
| `void` | `setMatrix(int i0, int i1, int j, DoubleVector v)`             Set the submatrix A[i0:i1][j] with the values stored in a DoubleVector |
| `void` | `setMatrix(int i0, int i1, int j0, int j1, double s)`             Set the submatrix A[i0:i1][j0:j1] with a same value |
| `void` | `setPlus(int i, int j, double s)`             Add a value to an element and reset the element |
| `void` | `setRowDimension(int rowDimension)`             Set the row dimenion of the matrix |
| `void` | `setTimes(int i, int j, double s)`             Multiply a value with an element and reset the element |
| `void` | `steplsqr(PaceMatrix b, IntVector pvt, int ks, int j, boolean adjoin)`             Stepwise least squares QR-decomposition of the problem A x = b |
| `double[]` | `sum2(boolean col)`             Squared sum of columns or rows of a matrix |
| `double` | `sum2(int j, int i0, int i1, boolean col)`             Squared sum of a column or row in a matrix |
| `double` | `times(int i, int j0, int j1, PaceMatrix B, int l)`             Multiplication between a row (or part of a row) of the first matrix and a column (or part or a column) of the second matrix. |
| `java.lang.String` | `toString()`             Converts matrix to string |
| `java.lang.String` | `toString(int digits, boolean trailing)`             Converts matrix to string |

**Methods inherited from class weka.core.matrix.Matrix**

`arrayLeftDivide, arrayLeftDivideEquals, arrayRightDivide, arrayRightDivideEquals, arrayTimes, arrayTimesEquals, chol, cond, constructWithCopy, copy, det, eig, get, getArray, getArrayCopy, getColumnDimension, getColumnPackedCopy, getMatrix, getMatrix, getMatrix, getMatrix, getRowDimension, getRowPackedCopy, identity, inverse, isSquare, isSymmetric, lu, minus, minusEquals, norm1, norm2, normF, normInf, parseMatlab, plus, plusEquals, print, print, print, print, qr, random, rank, read, regression, regression, set, setMatrix, setMatrix, setMatrix, setMatrix, solve, solveTranspose, sqrt, svd, times, times, timesEquals, toMatlab, trace, transpose, uminus, write`

**Methods inherited from class java.lang.Object**

`equals, getClass, hashCode, notify, notifyAll, wait, wait, wait`

**Constructor Detail**

### PaceMatrix

```
public PaceMatrix(int m,
                  int n)
```

:   Construct an m-by-n PACE matrix of zeros.

    **Parameters:**: `m` - Number of rows.: `n` - Number of colums.

---


### PaceMatrix

```
public PaceMatrix(int m,
                  int n,
                  double s)
```

:   Construct an m-by-n constant PACE matrix.

    **Parameters:**: `m` - Number of rows.: `n` - Number of colums.: `s` - Fill the matrix with this scalar value.

---


### PaceMatrix

```
public PaceMatrix(double[][] A)
```

:   Construct a PACE matrix from a 2-D array.

    **Parameters:**: `A` - Two-dimensional array of doubles. **Throws:**: `java.lang.IllegalArgumentException` - All rows must have the same length

---


### PaceMatrix

```
public PaceMatrix(double[][] A,
                  int m,
                  int n)
```

:   Construct a PACE matrix quickly without checking arguments.

    **Parameters:**: `A` - Two-dimensional array of doubles.: `m` - Number of rows.: `n` - Number of colums.

---


### PaceMatrix

```
public PaceMatrix(double[] vals,
                  int m)
```

:   Construct a PaceMatrix from a one-dimensional packed array

    **Parameters:**: `vals` - One-dimensional array of doubles, packed by columns (ala Fortran).: `m` - Number of rows. **Throws:**: `java.lang.IllegalArgumentException` - Array length must be a multiple of m.

---


### PaceMatrix

```
public PaceMatrix(DoubleVector v)
```

:   Construct a PaceMatrix with a single column from a DoubleVector

    **Parameters:**: `v` - DoubleVector

---


### PaceMatrix

```
public PaceMatrix(Matrix X)
```

:   Construct a PaceMatrix from a Matrix

    **Parameters:**: `X` - Matrix


**Method Detail**

### setRowDimension

```
public void setRowDimension(int rowDimension)
```

:   Set the row dimenion of the matrix

    :   **Parameters:**: `rowDimension` - the row dimension

---


### setColumnDimension

```
public void setColumnDimension(int columnDimension)
```

:   Set the column dimenion of the matrix

    :   **Parameters:**: `columnDimension` - the column dimension

---


### clone

```
public java.lang.Object clone()
```

:   Clone the PaceMatrix object.

    :   **Overrides:**: `clone` in class `Matrix`
    :   **Returns:**: the clone

---


### setPlus

```
public void setPlus(int i,
                    int j,
                    double s)
```

:   Add a value to an element and reset the element

    :   **Parameters:**: `i` - the row number of the element: `j` - the column number of the element: `s` - the double value to be added with

---


### setTimes

```
public void setTimes(int i,
                     int j,
                     double s)
```

:   Multiply a value with an element and reset the element

    :   **Parameters:**: `i` - the row number of the element: `j` - the column number of the element: `s` - the double value to be multiplied with

---


### setMatrix

```
public void setMatrix(int i0,
                      int i1,
                      int j0,
                      int j1,
                      double s)
```

:   Set the submatrix A[i0:i1][j0:j1] with a same value

    :   **Parameters:**: `i0` - the index of the first element of the column: `i1` - the index of the last element of the column: `j0` - the index of the first column: `j1` - the index of the last column: `s` - the value to be set to

---


### setMatrix

```
public void setMatrix(int i0,
                      int i1,
                      int j,
                      DoubleVector v)
```

:   Set the submatrix A[i0:i1][j] with the values stored in a
    DoubleVector

    :   **Parameters:**: `i0` - the index of the first element of the column: `i1` - the index of the last element of the column: `j` - the index of the column: `v` - the vector that stores the values

---


### setMatrix

```
public void setMatrix(double[] v,
                      boolean columnFirst)
```

:   Set the whole matrix from a 1-D array

    :   **Parameters:**: `v` - 1-D array of doubles: `columnFirst` - Whether to fill the column first or the row. **Throws:**: `java.lang.ArrayIndexOutOfBoundsException` - Submatrix indices

---


### maxAbs

```
public double maxAbs()
```

:   Returns the maximum absolute value of all elements

    :   **Returns:**: the maximum value

---


### maxAbs

```
public double maxAbs(int i0,
                     int i1,
                     int j)
```

:   Returns the maximum absolute value of some elements of a column,
    that is, the elements of A[i0:i1][j].

    :   **Parameters:**: `i0` - the index of the first element of the column: `i1` - the index of the last element of the column: `j` - the index of the column **Returns:**: the maximum value

---


### minAbs

```
public double minAbs(int i0,
                     int i1,
                     int column)
```

:   Returns the minimum absolute value of some elements of a column,
    that is, the elements of A[i0:i1][j].

    :   **Parameters:**: `i0` - the index of the first element of the column: `i1` - the index of the last element of the column: `column` - the index of the column **Returns:**: the minimum value

---


### isEmpty

```
public boolean isEmpty()
```

:   Check if the matrix is empty

    :   **Returns:**: true if the matrix is empty

---


### getColumn

```
public DoubleVector getColumn(int j)
```

:   Return a DoubleVector that stores a column of the matrix

    :   **Parameters:**: `j` - the index of the column **Returns:**: the column

---


### getColumn

```
public DoubleVector getColumn(int i0,
                              int i1,
                              int j)
```

:   Return a DoubleVector that stores some elements of a column of the
    matrix

    :   **Parameters:**: `i0` - the index of the first element of the column: `i1` - the index of the last element of the column: `j` - the index of the column **Returns:**: the DoubleVector

---


### times

```
public double times(int i,
                    int j0,
                    int j1,
                    PaceMatrix B,
                    int l)
```

:   Multiplication between a row (or part of a row) of the first matrix
    and a column (or part or a column) of the second matrix.

    :   **Parameters:**: `i` - the index of the row in the first matrix: `j0` - the index of the first column in the first matrix: `j1` - the index of the last column in the first matrix: `B` - the second matrix: `l` - the index of the column in the second matrix **Returns:**: the result of the multiplication

---


### toString

```
public java.lang.String toString()
```

:   Converts matrix to string

    :   **Overrides:**: `toString` in class `Matrix`
    :   **Returns:**: the matrix as string

---


### toString

```
public java.lang.String toString(int digits,
                                 boolean trailing)
```

:   Converts matrix to string

    :   **Parameters:**: `digits` - number of digits after decimal point: `trailing` - true if trailing zeros are padded **Returns:**: the matrix as string

---


### sum2

```
public double sum2(int j,
                   int i0,
                   int i1,
                   boolean col)
```

:   Squared sum of a column or row in a matrix

    :   **Parameters:**: `j` - the index of the column or row: `i0` - the index of the first element: `i1` - the index of the last element: `col` - if true, sum over a column; otherwise, over a row **Returns:**: the squared sum

---


### sum2

```
public double[] sum2(boolean col)
```

:   Squared sum of columns or rows of a matrix

    :   **Parameters:**: `col` - if true, sum over columns; otherwise, over rows **Returns:**: the squared sum

---


### h1

```
public double[] h1(int j,
                   int k)
```

:   Constructs single Householder transformation for a column

    :   **Parameters:**: `j` - the index of the column: `k` - the index of the row **Returns:**: d and q

---


### h2

```
public void h2(int j,
               int k,
               double q,
               PaceMatrix b,
               int l)
```

:   Performs single Householder transformation on one column of a matrix

    :   **Parameters:**: `j` - the index of the column: `k` - the index of the row: `q` - q = - u'u/2; must be negative: `b` - the matrix to be transformed: `l` - the column of the matrix b

---


### g1

```
public double[] g1(double a,
                   double b)
```

:   Constructs the Givens rotation

    :   **Parameters:**: `a` -: `b` - **Returns:**: a double array that stores the cosine and sine values

---


### g2

```
public void g2(double[] cs,
               int i0,
               int i1,
               int j)
```

:   Performs the Givens rotation

    :   **Parameters:**: `cs` - a array storing the cosine and sine values: `i0` - the index of the row of the first element: `i1` - the index of the row of the second element: `j` - the index of the column

---


### forward

```
public void forward(PaceMatrix b,
                    IntVector pvt,
                    int k0)
```

:   Forward ordering of columns in terms of response explanation. On
    input, matrices A and b are already QR-transformed. The indices of
    transformed columns are stored in the pivoting vector.

    :   **Parameters:**: `b` - the PaceMatrix b: `pvt` - the pivoting vector: `k0` - the first k0 columns (in pvt) of A are not to be changed

---


### mostExplainingColumn

```
public int mostExplainingColumn(PaceMatrix b,
                                IntVector pvt,
                                int ks)
```

:   Returns the index of the column that has the largest (squared)
    response, when each of columns pvt[ks:] is moved to become the
    ks-th column. On input, A and b are both QR-transformed.

    :   **Parameters:**: `b` - response: `pvt` - pivoting index of A: `ks` - columns pvt[ks:] of A are to be tested **Returns:**: the index of the column

---


### backward

```
public void backward(PaceMatrix b,
                     IntVector pvt,
                     int ks,
                     int k0)
```

:   Backward ordering of columns in terms of response explanation. On
    input, matrices A and b are already QR-transformed. The indices of
    transformed columns are stored in the pivoting vector.
    A and b must have the same number of rows, being the (pseudo-)rank.

    :   **Parameters:**: `b` - PaceMatrix b: `pvt` - pivoting vector: `ks` - number of QR-transformed columns; psuedo-rank of A: `k0` - first k0 columns in pvt[] are not to be ordered.

---


### leastExplainingColumn

```
public int leastExplainingColumn(PaceMatrix b,
                                 IntVector pvt,
                                 int ks,
                                 int k0)
```

:   Returns the index of the column that has the smallest (squared)
    response, when the column is moved to become the (ks-1)-th
    column. On input, A and b are both QR-transformed.

    :   **Parameters:**: `b` - response: `pvt` - pivoting index of A: `ks` - psudo-rank of A: `k0` - A[][pvt[0:(k0-1)]] are excluded from the testing. **Returns:**: the index of the column

---


### columnResponseExplanation

```
public double columnResponseExplanation(PaceMatrix b,
                                        IntVector pvt,
                                        int j,
                                        int ks)
```

:   Returns the squared ks-th response value if the j-th column becomes
    the ks-th after orthogonal transformation. A[][pvt[ks:j]] (or
    A[][pvt[j:ks]], if ks > j) and b[] are already QR-transformed
    on input and will remain unchanged on output.
    More generally, it returns the inner product of the corresponding
    row vector of the response PaceMatrix. (To be implemented.)

    :   **Parameters:**: `b` - PaceMatrix b: `pvt` - pivoting vector: `j` - the column A[pvt[j]][] is to be moved: `ks` - the target column A[pvt[ks]][] **Returns:**: the squared response value

---


### lsqr

```
public void lsqr(PaceMatrix b,
                 IntVector pvt,
                 int k0)
```

:   QR transformation for a least squares problem

---


### lsqrSelection

```
public void lsqrSelection(PaceMatrix b,
                          IntVector pvt,
                          int k0)
```

:   QR transformation for a least squares problem

---


### positiveDiagonal

```
public void positiveDiagonal(PaceMatrix Y,
                             IntVector pvt)
```

:   Sets all diagonal elements to be positive (or nonnegative) without
    changing the least squares solution

    :   **Parameters:**: `Y` - the response: `pvt` - the pivoted column index

---


### steplsqr

```
public void steplsqr(PaceMatrix b,
                     IntVector pvt,
                     int ks,
                     int j,
                     boolean adjoin)
```

:   Stepwise least squares QR-decomposition of the problem
    A x = b

    :   **Parameters:**: `b` - PaceMatrix b: `pvt` - pivoting vector: `ks` - number of transformed columns: `j` - pvt[j], the column to adjoin or delete: `adjoin` - to adjoin if true; otherwise, to delete

---


### rsolve

```
public void rsolve(PaceMatrix b,
                   IntVector pvt,
                   int kp)
```

:   Solves upper-triangular equation

---


### rbind

```
public PaceMatrix rbind(PaceMatrix b)
```

:   Returns a new matrix which binds two matrices together with rows.

    :   **Parameters:**: `b` - the second matrix **Returns:**: the combined matrix

---


### cbind

```
public PaceMatrix cbind(PaceMatrix b)
```

:   Returns a new matrix which binds two matrices with columns.

    :   **Parameters:**: `b` - the second matrix **Returns:**: the combined matrix

---


### nnls

```
public DoubleVector nnls(PaceMatrix b,
                         IntVector pvt)
```

:   Solves the nonnegative linear squares problem. That is,

    min || A x - b||, subject to x >= 0. 

    For algorithm, refer to P161, Chapter 23 of C. L. Lawson and
    R. J. Hanson (1974). "Solving Least Squares
    Problems". Prentice-Hall.

    :   **Parameters:**: `b` - the response: `pvt` - vector storing pivoting column indices **Returns:**: solution

---


### nnlse

```
public DoubleVector nnlse(PaceMatrix b,
                          PaceMatrix c,
                          PaceMatrix d,
                          IntVector pvt)
```

:   Solves the nonnegative least squares problem with equality
    constraint. That is,

    min ||A x - b||, subject to x >= 0 and c x = d. 

    :   **Parameters:**: `b` - the response: `c` - coeficients of equality constraints: `d` - constants of equality constraints: `pvt` - vector storing pivoting column indices **Returns:**: the solution

---


### nnlse1

```
public DoubleVector nnlse1(PaceMatrix b,
                           IntVector pvt)
```

:   Solves the nonnegative least squares problem with equality
    constraint. That is,

    min ||A x - b||, subject to x >= 0 and || x || = 1. 

    :   **Parameters:**: `b` - the response: `pvt` - vector storing pivoting column indices **Returns:**: the solution

---


### randomNormal

```
public static Matrix randomNormal(int m,
                                  int n)
```

:   Generate matrix with standard-normally distributed random elements

    :   **Parameters:**: `m` - Number of rows.: `n` - Number of colums. **Returns:**: An m-by-n matrix with random elements.

---


### getRevision

```
public java.lang.String getRevision()
```

:   Returns the revision string.

    :   **Specified by:**: `getRevision` in interface `RevisionHandler` **Overrides:**: `getRevision` in class `Matrix`
    :   **Returns:**: the revision

---


### main

```
public static void main(java.lang.String[] args)
```

:   for testing only

    :   **Parameters:**: `args` - the commandline arguments - ignored


---


|  |  |  |
| --- | --- | --- |
| **Overview** \| **Package** \| **Class** \| **Tree** \| **Deprecated** \| **Index** \| **Help** \| **Weka's home** | |  |
| **PREV CLASS**   NEXT CLASS | **FRAMES**    **NO FRAMES**     **All Classes** |  |
| SUMMARY: NESTED \| FIELD \| CONSTR \| METHOD | DETAIL: FIELD \| CONSTR \| METHOD |  |


---
